# Supplementary material for: Learning curve and surgical outcome of robotic assisted colorectal surgery with ERAS program
Source: Sci Rep. 2022 Nov 29;12:20566. doi: 10.1038/s41598-022-24665-w (PMC9709162; doi:10.1038/s41598-022-24665-w)
Supplement: Supplementary file 1 — Supplementary Information 1. [file 41598_2022_24665_MOESM1_ESM.zip › LSC Raw data-Ñ[▒K/105/P_ñw╝╨░O▒Kñσ.pdf]

臺中榮民總醫院 臺中市臺灣大道四段1650號 病理部活體組織診斷報告 臨床醫學實驗及病理診斷中心 檢驗(查)報告

[redacted]

Pathologic diagnosis:
1. Colon, transverse to sigmoid, laparoscopic anterior resection --- Status post endoscopic mucosal resection with submucosal hemorrhage. No residual tumor is found. The bilateral cut ends and mesocolic lymph nodes (0/9) are free.
2. Colon, cut end, distal, resection --- Chronic inflammation.

Gross description:
The specimen consists of 1) a segment of colon, 17 cm in length and 4.5 cm in proximal cut end circumference, 4 cm in distal circumference, and mesocolic soft tissue. Focal erythematous changes over the mucosa is noted. No mass lesion is identified grossly. The both cut ends are free of lesion grossly. 2) distal cut end, a piece of tan mucosa-covered soft tissue, 3x1.8x1.1 cm. Representative part for section: A) proximal cut end B) distal cut end C-E) erythematous mucosa F) specimen 2 G-K) lymph nodes.
#T-59300_2 #M-43000_2 0 1105 SP0000
#T-C4560_2 #M-09183_2 0 1105 000000

已電子簽章

敬告：本單僅供醫師參考使用，不具任何申請效力！！
請病患妥為保管，若不慎遺失需自負個人資料外洩責任！
